# Associations between a body shape index and coronary heart disease: a case–control study in southern China

**DOI:** 10.3389/fcvm.2026.1698541

**Published:** 2026-02-04

**Authors:** Weikun Zhao, Ruiyan Huang, Renxuan Qin, Xinlong Zhang, Jinquan Zeng, Feng Huang, Rongjie Huang

**Affiliations:** 1 Department of Cardiovascular Medicine, Third Ward, The First Affiliated Hospital of Guangxi Medical University, Nanning, Guangxi, China; 2 Premium Healthcare Department, The First Affiliated Hospital of Guilin Medical University, Guilin, Guangxi, China; 3 Guangxi Key Laboratory of Precision Medicine in Cardio-Cerebrovascular Diseases Control and Prevention, The First Affiliated Hospital of Guangxi Medical University, Nanning, Guangxi, China; 4 Guangxi Clinical Research Center for Cardio-Cerebrovascular Diseases, The First Affiliated Hospital of Guangxi Medical University, Nanning, Guangxi, China

**Keywords:** a body shape index, case–control study, central obesity, coronary heart disease, risk factors

## Abstract

**Background:**

Coronary heart disease (CHD) burden is increasing, and traditional obesity measures inadequately capture fat distribution and associated CHD risk. A body shape index (ABSI) is an emerging anthropometric metric of fat distribution, but evidence linking ABSI to CHD is limited, particularly in the Chinese population. This case-control study in southern China investigated the association of ABSI and related factors with CHD risk, aiming to facilitate early identification of high-risk individuals.

**Methods:**

We retrospectively studied 996 patients who underwent coronary angiography in a southern Chinese hospital. After strict screening and propensity score matching (PSM), 125 patients with CHD (>50% coronary stenosis) and 125 controls (<50% stenosis) were selected. Key CHD risk predictors were identified using feature-selection techniques (LASSO regression, recursive feature elimination, random forest importance). Univariate and multivariate logistic regression models were constructed for CHD prediction. Model performance was evaluated by receiver operating characteristic (ROC) analysis and compared to individual predictors using the DeLong test. A nomogram was developed for individualized risk estimation.

**Results:**

Baseline characteristics were well matched between CHD and control groups after PSM. Across feature-selection methods, the most influential predictors for CHD included ABSI, prealbumin (PA), direct-to-total bilirubin ratio (DB/TB), apolipoprotein B (ApoB), globulin (GLO), apolipoprotein A-I (ApoA-I), and essential hypertension (EH). Each of these factors showed a significant univariate association with CHD (*P* < 0.05) but only modest predictive power individually (AUCs 0.57–0.66). ABSI exhibited the highest sensitivity (86.4%) among single predictors, while ApoB had the highest specificity (78.4%). The multivariable logistic model incorporating these variables achieved an AUC of 0.809, significantly outperforming any individual predictor (*P* < 0.001). At the optimal probability cutoff, the model's sensitivity and specificity were 69.6% and 82.4%, respectively. The nomogram combined ABSI with other key variables to provide a quantitative CHD risk estimate for individual patients.

**Conclusions:**

This study identifies ABSI as a potential predictor of CHD risk among southern Chinese populations. Integrating ABSI with other candidate predictors improves the model's predictive performance. A multifactorial approach may better characterize CHD risk in this population and could inform prevention strategies.

## 1 Introduction

Coronary heart disease (CHD) is the leading cause of death worldwide. In 2022 alone, roughly 19.8 million people died from cardiovascular diseases—about one third of all deaths—with 85% attributed to myocardial infarction or stroke; more than three-quarters of these deaths occurred in low- and middle-income countries (1). The burden of cardiovascular disease in China has been steadily increasing, with a growing number of cases and deaths. An estimated 330 million people are affected, and in 2019 cardiovascular conditions accounted for 46.74% of all deaths in rural areas and 44.26% in urban areas (2). From 2013 to 2021, deaths due to heart disease and their contribution to total mortality increased significantly, and heart disease became the leading cause of death, consistent with a growing CHD-related mortality burden (3). The rapidly developing southern regions bear an even heavier burden of CHD, driven by more calorie-dense diets, sedentary habits and population ageing (4). Identifying CHD risk factors is therefore crucial for devising effective prevention and intervention strategies.

Traditional CHD risk factors include older age, male sex, smoking, hypertension, and dyslipidemia (5). Among these, obesity is an important modifiable factor, and in particular, central (abdominal) obesity, characterized by excess visceral fat, has been shown to have a significant association with increased risk of cardiovascular events (6, 7). However, the commonly used body mass index (BMI) cannot capture differences in fat distribution. Indeed, individuals with higher abdominal fat at the same BMI have greater metabolic and cardiovascular risk than those with fat stored peripherally (8). Thus, new indicators are needed to improve CHD risk assessment.

The a body shape index (ABSI) combines waist circumference, height and BMI. Developed through allometric regression to be independent of BMI, it specifically captures central adiposity (9). Growing evidence shows that ABSI is strongly associated with cardiometabolic risk factors and metabolic syndrome (9, 10), and it can independently predict mortality. For example, in the US National Health and Nutrition Examination Survey cohort, each one-standard-deviation increase in ABSI—after adjusting for traditional factors—was associated with a substantial increase in CHD risk (odds ratio ≈ 1.31) (11). Compared with BMI, ABSI better reflects visceral fat accumulation and thus offers a distinctive advantage in assessing CHD risk (9, 12).

Although numerous studies on ABSI have been conducted in Western populations (8, 12–15), relevant evidence for Chinese populations, particularly those in southern regions, remains relatively limited. Some studies in China have begun to explore the relationship between ABSI and cardiovascular disease risk (16, 17), highlighting ABSI's independent predictive value in normal-weight individuals and comparing its predictive performance with other obesity indices. However, in-depth region-specific studies focusing on the association between ABSI and CHD risk in southern Chinese populations are still lacking. Evidence suggests that Asians (including Chinese) tend to accumulate more abdominal fat at lower BMI levels, which may affect CHD risk assessment (4, 18, 19). In addition, the dietary habits, lifestyle, and genetic background of southern Chinese populations differ from those of other groups, potentially contributing to regional variations in CHD risk factors. To fill this regional research gap, we carried out a case–control study in southern China with the following aims: (1) to evaluate the association between ABSI and the risk of CHD; (2) to control for confounding through propensity score matching (PSM) and construct a multivariable model integrating ABSI, apolipoprotein B (ApoB), globulin (GLO), and other indices to comprehensively assess their predictive value for CHD risk; (3) to develop a nomogram for individualized risk prediction. Compared to previous studies, the innovation of this work lies in the first application of a PSM-matched case–control design in a southern Chinese population, integrating multiple factors into a single model and providing a visualization tool. This approach provides practical guidance for CHD risk screening and precision prevention strategies in this population.

## 2 Materials and methods

### 2.1 Study design and grouping

**Figure 1:**
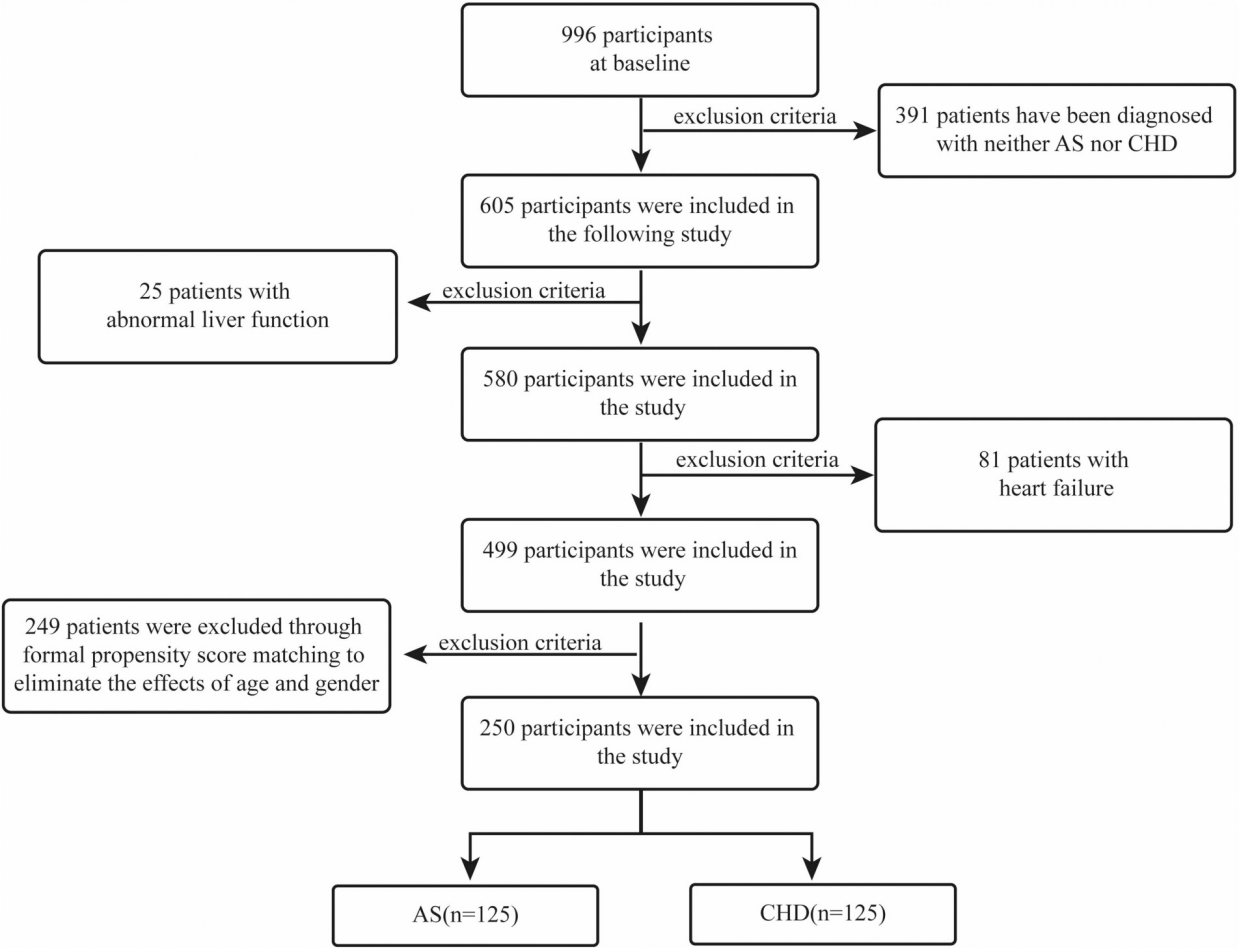
Participant selection flow diagram.

We conducted a retrospective case-control investigation of patients who underwent coronary angiography in the Department of Cardiology, First Affiliated Hospital of Guangxi Medical University, between January 2019 and January 2021. According to coronary angiographic classification, participants were grouped into an atherosclerosis (AS) comparison group and a CHD group. The inclusion criteria were as follows: (i) CHD group-coronary angiography showing ≥50% coronary stenosis; (ii) AS group-coronary angiography showing <50% coronary stenosis. Exclusion criteria included: (i) patients diagnosed with acute myocardial infarction; (ii) patients with acute or chronic heart failure; (iii) patients with liver diseases (AST > 80 U/L or ALT > 80 U/L). To curb confounding, we applied stringent eligibility filters followed by 1:1 PSM between CHD and AS cohorts, yielding 125 patients per arm (Figure 1). This design and matching approach ensured baseline comparability between the CHD and AS groups, thereby reducing potential confounding bias and improving the robustness of the results. Specifically, 996 baseline subjects were initially enrolled. After excluding individuals without CHD or AS diagnoses, 605 participants were retained for the study. Following further exclusions of 25 patients with abnormal liver function and 81 patients with heart failure, a final cohort of 499 participants was included in the analysis. To account for the effects of age and sex, PSM was used, ensuring the final matched groups consisted of 125 participants each from both the AS and CHD groups.

This study was approved by the Ethics Committee of the First Affiliated Hospital of Guangxi Medical University (ethical approval number: 2024-E706-01) and conducted in accordance with the Helsinki Declaration. The patients provided written informed consent to participate in this study.

### 2.2 Baseline characteristics and variable selection

Our baseline characteristics included sex, age, BMI, waist circumference, diabetes mellitus (DM) status, essential hypertension (EH) status, lipid profiles and related metabolic parameters. Blood pressure was measured twice using an automated electronic sphygmomanometer and the average was recorded. Hypertension was defined as a systolic pressure ≥140 mmHg and/or diastolic pressure ≥90 mmHg, or current antihypertensive therapy. Diabetes mellitus was defined according to American Diabetes Association criteria: fasting glucose ≥7.0 mmol/L, 2-hour post-load plasma glucose ≥11.1 mmol/L on an oral glucose tolerance test, or haemoglobin A1c ≥6.5%. In our study, EH and DM were ascertained using comprehensive electronic medical record data rather than self-report, including: (i) prior diagnostic notes; (ii) objective readings from hospital exams or stays, like blood pressure or glucose levels; (iii) history of relevant medications, such as prescriptions for blood pressure or diabetes drugs.

**Figure 2:**
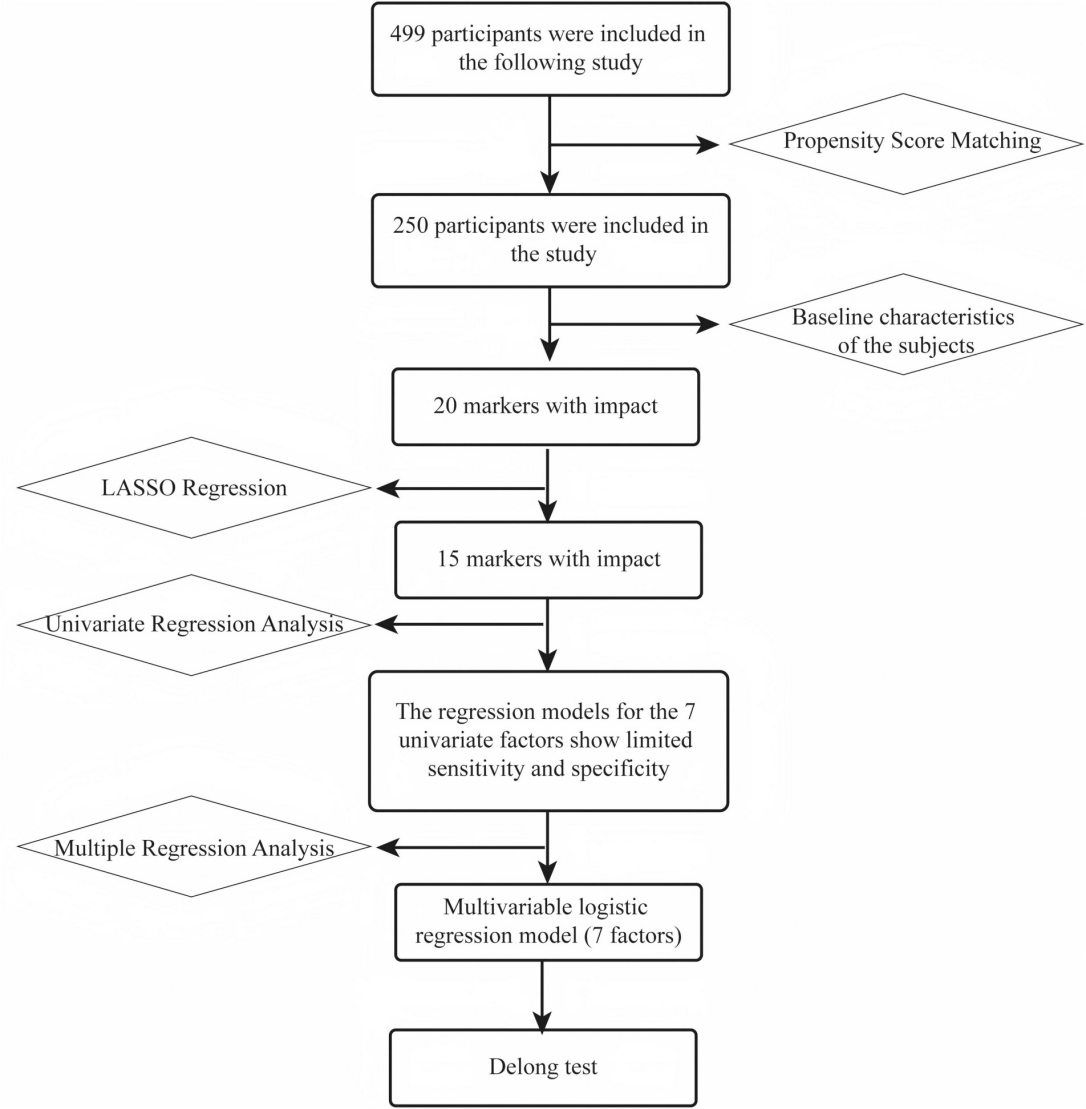
Analytical workflow and variable selection.

Baseline characteristics were first compared in the matched sample to identify 20 “influential” indicators. Subsequently, using LASSO regression and recursive feature elimination (RFE), the candidate features were reduced to 15 significant indicators. These 15 variables were then analyzed using univariate regression, resulting in 7 individual models. Based on these 7 factors, a multivariable logistic regression model was constructed, and the model performance was compared using DeLong's test (Figure 2).

### 2.3 Coronary angiography assessment

Two senior cardiovascular specialists independently evaluated the coronary angiography results in this study. In cases of disagreement between the two experts, a third cardiovascular physician was consulted for arbitration. To minimize intra-observer variability, all evaluations were conducted within the same time frame whenever possible. The coronary angiography assessment included evaluation of the left main coronary artery, right coronary artery, left anterior descending artery, and left circumflex artery for stenotic lesions. Lesions with ≥50% stenosis were considered clinically significant. Based on the number of affected vessels, patients were further categorized into the following groups: single-vessel disease, double-vessel disease, and triple-vessel disease.

### 2.4 Laboratory testing

All patients’ biochemical parameters were measured using an automated biochemical analyzer, including fasting blood glucose (FBG), total cholesterol (TC), triglycerides (TG), low-density lipoprotein cholesterol (LDL-C), high-density lipoprotein cholesterol (HDL-C), apolipoprotein A-I (ApoA-I), ApoB, total protein (TP), albumin (ALB), GLO, albumin/globulin ratio (A/G), prealbumin (PA), serum alanine aminotransferase (ALT), aspartate aminotransferase (AST), serum creatinine (SCr), and blood urea nitrogen (BUN). All blood samples were collected via venipuncture after an 8-hour fast and immediately sent for testing to ensure result accuracy. Blood glucose levels were used to assess glycemic status; lipid parameters (TC, TG, LDL-C, HDL-C, ApoA-I, ApoB) to assess lipid metabolism; ALT and AST to evaluate liver function; SCr and BUN to evaluate renal function; and the A/G ratio to evaluate protein metabolism and systemic inflammatory status. ABSI was calculated using the standard formula proposed by Krakauer et al. (8): ABSI = WC/(BMI^2/3^ × H^1/2^), where WC stands for waist circumference (m), BMI for body mass index (kg/m²), and H for height (m). All tests were performed according to standard operating procedures to ensure data reliability and reproducibility.

### 2.5 Statistical methods

Statistical analysis was performed using RStudio (version R 4.4.1), with *P* < 0.05 considered statistically significant. Continuous variables were expressed as mean ± standard deviation (mean ± SD), and categorical variables as counts and percentages. The normality of data was evaluated by the Kolmogorov–Smirnov test. For normally distributed continuous variables, an independent-sample *t* test was used for between-group comparisons; for categorical variables, the *χ*² test or Fisher's exact test was applied. Non-normally distributed continuous variables were expressed as median, and the Mann–Whitney *U* test was used for between-group comparisons. Through strict screening and PSM, 250 participants were included to balance baseline characteristics and reduce selection bias.

Statistical analysis proceeded as follows: key variables were selected from 20 potential risk factors using least absolute shrinkage and selection operator (LASSO) regression and random forest analysis. LASSO regression, employing L1 regularization and 10-fold cross-validation to determine the optimal penalty parameter *λ* [log(*λ*) ≈ −4] and combined with recursive feature elimination, was used to optimize the variable set, ultimately identifying seven key factors [ABSI, direct-to-total bilirubin ratio (DB/TB), PA, GLO, ApoA-I, ApoB, and EH]. Each selected variable was subjected to univariate logistic regression to calculate odds ratios (ORs) and 95% confidence intervals; receiver operating characteristic (ROC) curves were plotted to compute area under the curve (AUC) values, and the Youden index was used to determine the optimal cutoff values along with corresponding sensitivities and specificities. A multivariable logistic regression model was constructed using the seven key variables and its AUC was calculated. The AUCs of the multivariable model and the univariate models were compared using the DeLong test to evaluate the advantage of the multivariable model. Variable importance was assessed via random forest analysis to identify the factors that contributed most to prediction. Finally, a nomogram based on the multivariable model was developed for clinical prediction of individual CHD risk.

## 3 Results

### 3.1 Comparison before and after PSM matching

Before matching, the CHD and AS groups differed significantly in multiple variables (such as sex, age; *P* < 0.05). After matching, differences in key demographic variables (e.g., age, sex) were greatly reduced and no longer statistically significant (*P* > 0.05) between the groups, but several metabolic, hematological, and anthropometric parameters (such as EH, DB/TB, GLO, A/G, PA, ApoA-I, ApoB) still exhibited significant differences (*P* < 0.05). These residual imbalances were retained as candidate predictors for subsequent feature selection. Notably, ABSI remained significantly different after matching (AS group 0.00754 ± 0.000674 vs. CHD group 0.00789 ± 0.000620, *P* < 0.001) (Table 1).

**Table 1:** Baseline characteristics comparison between AS group and CHD group before and after matching.

Characteristics	Before matched		*P* value	After matched		*P* value
	AS (*n* = 143)	CHD (*n* = 356)		AS (*n* = 125)	CHD (*n* = 125)	
Sex						
Female (*n*, %)	72 (50.30%)	141 (39.60%)	0.036	57 (45.60%)	63 (50.40%)	0.527
Male (*n*, %)	71 (49.70%)	215 (60.40%)		68 (54.40%)	62 (49.60%)	
Age, years (mean, SD)	62.4 (11.40)	58.5 (14.10)	0.002	61.0 (11.10)	59.7 (14.70)	0.447
Height, cm (mean, SD)	161 (7.84)	162 (8.96)	0.129	162 (7.74)	160 (8.18)	0.110
Weight, cm (mean, SD)	63.7 (12.70)	64.6 (13.10)	0.479	64.5 (12.80)	62.5 (11.40)	0.184
Waist, cm (mean, SD)	80.7 (10.70)	84.6 (10.40)	<0.001	80.6 (11.20)	83.3 (9.52)	0.043
BMI, kg/m² (mean, SD)	24.4 (3.62)	24.4 (3.75)	0.967	24.5 (3.66)	24.2 (3.47)	0.584
DM						
No	127 (88.80%)	284 (79.80%)	0.024	112 (89.60%)	99 (79.20%)	0.037
Yes	16 (11.20%)	72 (20.20%)		13 (10.40%)	26 (20.80%)	
EH						
No	59 (41.30%)	72 (20.20%)	<0.001	48 (38.40%)	29 (23.20%)	0.014
Yes	84 (58.70%)	284 (79.80%)		77 (61.60%)	96 (76.80%)	
DBiL, μmol/L (mean, SD)	4.14 (2.02)	4.13 (2.14)	0.964	4.20 (2.11)	4.38 (2.31)	0.510
IBiL, μmol/L (mean, SD)	10.2 (6.53)	8.88 (4.97)	0.025	10.5 (6.81)	8.99 (4.90)	0.047
DB/TB (mean, SD)	0.301 (0.08)	0.329 (0.10)	<0.001	0.300 (0.08)	0.335 (0.10)	0.002
TP, g/L (mean, SD)	69.8 (6.12)	66.9 (6.05)	<0.001	70.1 (6.10)	67.0 (5.47)	<0.001
ALB, g/L (mean, SD)	38.3 (3.79)	38.4 (3.65)	0.805	38.5 (3.54)	38.1 (3.55)	0.323
GLO, g/L (mean, SD)	31.5 (5.49)	28.5 (4.62)	<0.001	31.6 (5.74)	28.9 (4.34)	<0.001
A/G (mean, SD)	1.25 (0.24)	1.38 (0.25)	<0.001	1.26 (0.24)	1.34 (0.23)	0.005
AST, U/L (mean, SD)	27.5 (10.50)	25.3 (9.06)	0.029	27.9 (11.00)	25.0 (8.59)	0.022
ALT, U/L (mean, SD)	20.1 (11.60)	22.0 (13.40)	0.116	20.7 (11.90)	21.0 (13.90)	0.830
AST/ALT (mean, SD)	1.63 (0.87)	1.46 (0.96)	0.051	1.57 (0.76)	1.53 (1.00)	0.717
ALP, U/L (mean, SD)	71.7 (23.80)	76.9 (23.30)	0.028	72.3 (24.30)	77.0 (22.10)	0.115
UA, μmol/L (mean, SD)	358 (106)	390 (119)	0.003	364 (108)	379 (116)	0.311
ApoA-I, g/L (mean, SD)	1.32 (0.23)	1.21 (0.22)	<0.001	1.32 (0.24)	1.22 (0.24)	0.001
ApoB, g/L (mean, SD)	0.867 (0.20)	0.930 (0.26)	0.004	0.861 (0.20)	0.924 (0.25)	0.028
ApoA/B (mean, SD)	1.61 (0.48)	1.39 (0.49)	<0.001	1.61 (0.49)	1.41 (0.56)	0.003
MCHC, g/L (mean, SD)	328 (11.50)	331 (12.70)	0.007	328 (11.90)	332 (12.60)	0.015
RDWCV, % (mean, SD)	0.146 (0.05)	0.169 (0.09)	<0.001	0.146 (0.05)	0.162 (0.08)	0.045
HCT, % (mean, SD)	0.390 (0.07)	0.366 (0.09)	0.002	0.393 (0.07)	0.366 (0.09)	0.006
HbA1a, % (mean, SD)	0.538 (0.14)	0.595 (0.17)	<0.001	0.546 (0.13)	0.592 (0.15)	0.009
HbA1b, % (mean, SD)	0.775 (0.19)	0.731 (0.19)	0.020	0.776 (0.17)	0.730 (0.19)	0.049
VAI (mean, SD)	114 (94)	123 (308)	0.602	114 (96.20)	105 (73.30)	0.443
BRI (mean, SD)	19.4 (0.07)	19.4 (0.06)	0.002	19.4 (0.06)	19.4 (0.06)	0.010
ABSI (mean, SD)	0.00758 (0.000683)	0.00793 (0.000639)	<0.001	0.00754 (0.000674)	0.00789 (0.000620)	<0.001

### 3.2 LASSO regression and random forest variable selection

**Figure 3:**
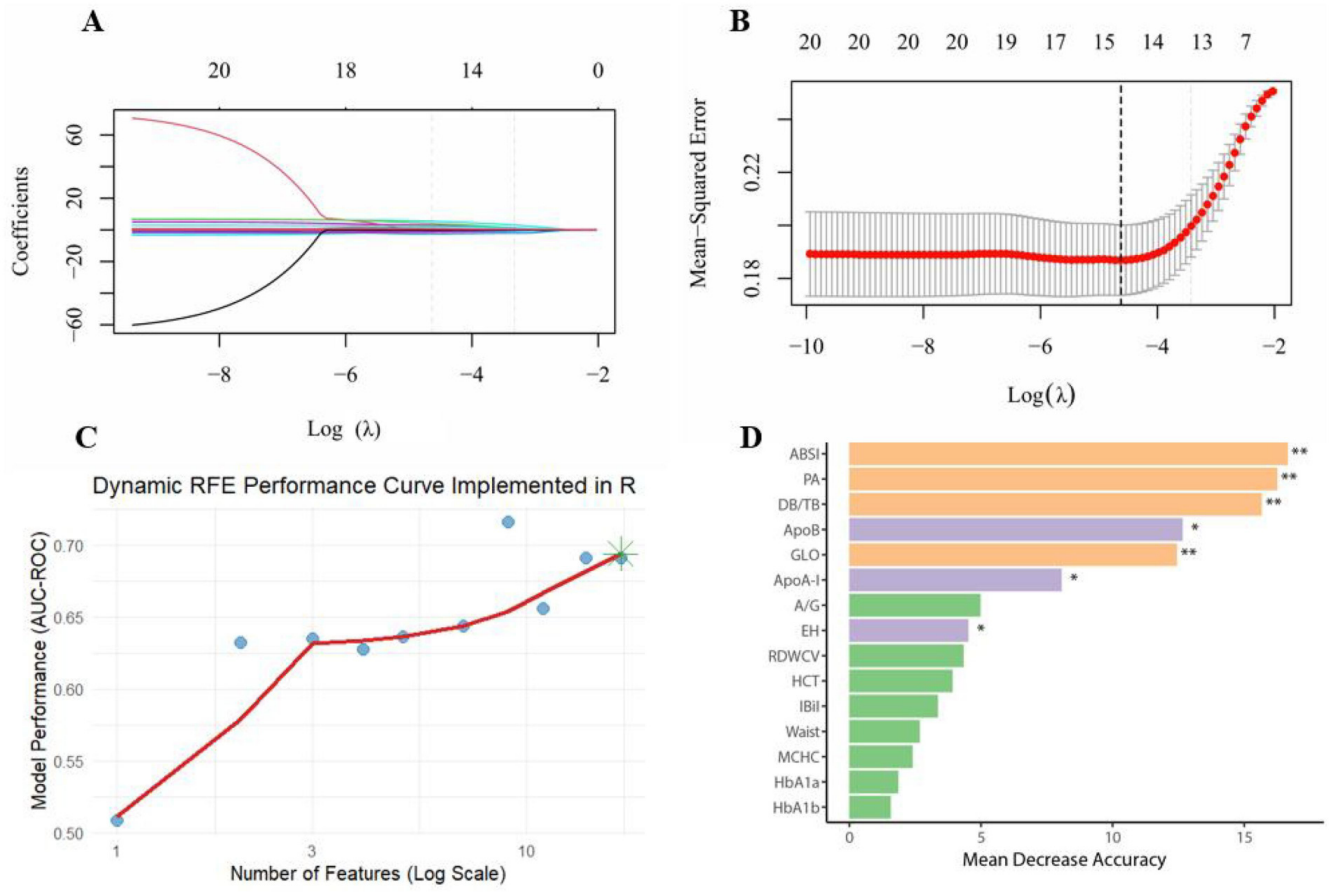
Multi-method feature selection and validation for CHD risk modelling. **(A)** LASSO coefficient paths; **(B)** Ten-fold cross-validation; **(C)** Dynamic RFE performance curve; **(D)** Variable importance in the random forest; **P < 0.01, *P < 0.05.

In this study, LASSO regression and random forest were used to select and assess the importance of CHD risk factors. As log(*λ*) increased from approximately −10 to −2 in LASSO regression, the coefficients of most variables gradually shrank towards zero (Figure 3A). A few variables exhibited large positive or negative coefficients with smaller penalties, which rapidly diminished as the penalty increased, while the rest of the variables remained close to zero. The cross-validation error curve identified the optimal regularization parameter *λ*, where the model achieved the best variable selection and predictive performance, retaining 15 non-zero coefficients (Figure 3B). With dynamic RFE, the AUC–ROC curve plotted against feature count on a logarithmic scale showed an overall upward trend; the subset highlighted by the green star at the rightmost end achieved the highest model performance (Figure 3C). Random forest importance ranking independently validated the predictive efficacy of these key variables, with variable importance ranked from highest to lowest as follows: ABSI, PA, DB/TB, ApoB, GLO, ApoA-I, A/G, EH, RDWCV, HCT, IBiL, Waist, MCHC, HbA1a, HbA1b (Figure 3D). Among these, ABSI was ranked the highest in terms of importance, and removing variables like PA, the bilirubin ratio, lipoprotein-related markers, and GLO would also cause a notable drop in the model's accuracy.

### 3.3 Evaluation of key predictors in univariate analysis

**Figure 4:**
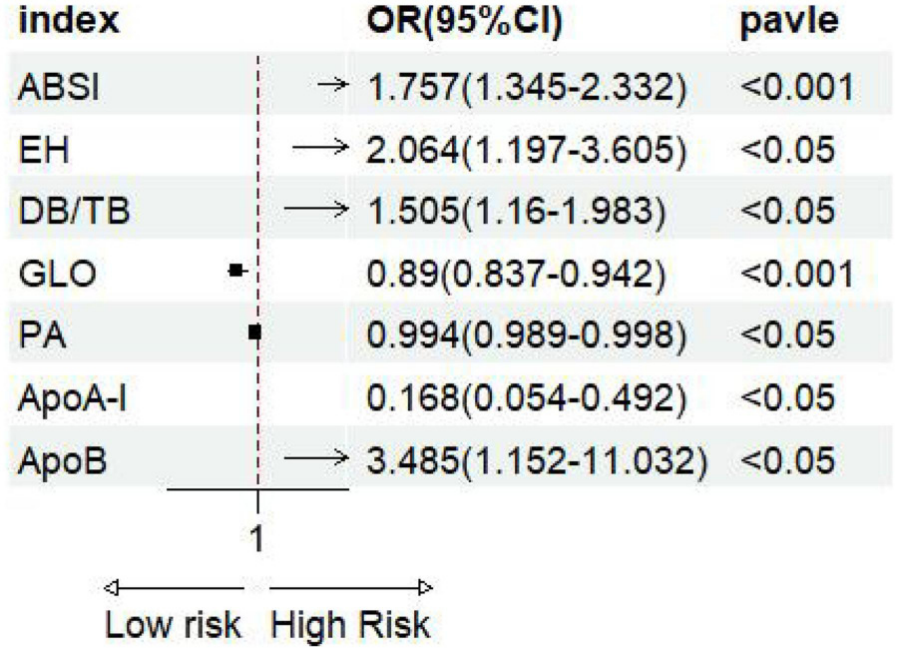
Forest plot of univariate logistic regression analysis.

**Figure 5:**
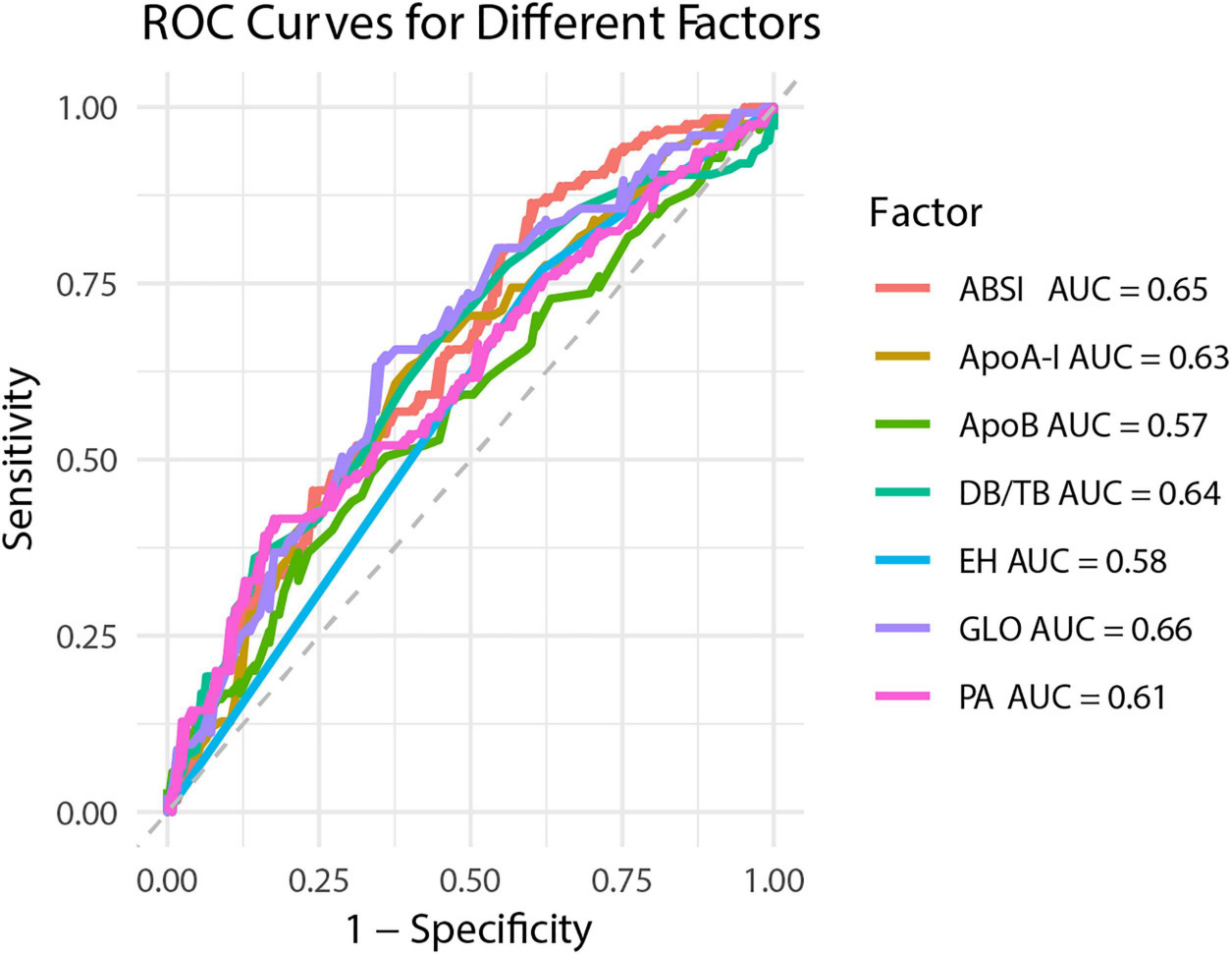
ROC curves for univariate regression analysis.

The forest plot of univariate binary logistic regression (Figure 4) indicated that ABSI (OR = 1.757, 95% CI: 1.345–2.332, *P* < 0.001), EH (OR = 2.064, 95% CI: 1.197–3.605, *P* < 0.05) and ApoB (OR = 3.485, 95% CI: 1.152–11.032, *P* < 0.05) were significant risk factors (marked by arrows), whereas GLO (OR = 0.89), PA (OR = 0.994) and ApoA-I (OR = 0.168) served as protective factors (all *P* < 0.05). The ROC curves for all indicators lay above the diagonal reference line, indicating some discriminative power (Figure 5). The AUC values were as follows: GLO 0.66, ABSI 0.65, DB/TB 0.64, ApoA-I 0.63, PA 0.61, EH 0.58 and ApoB 0.57. Optimal cutoff values, representing the predicted probabilities from the logistic regression model, and corresponding sensitivities and specificities from Table 2 were: ABSI threshold 42.0% (sensitivity 86.4%, specificity 40.0%); EH threshold 46.6% (76.8%, 38.4%); DB/TB threshold 47.5% (68.0%, 54.4%); GLO threshold 52.2% (64.8%, 64.0%); PA threshold 54.2% (41.6%, 82.4%); ApoA-I threshold 50.5% (63.2%, 60.0%); and ApoB threshold 52.9% (36.8%, 78.4%).

**Table 2:** Sensitivity and specificity of ROC curves for univariate logistic regression analysis.

Factor	Threshold	Sensitivity	Specificity
ABSI	42.0%	86.4%	40.0%
EH	46.6%	76.8%	38.4%
DB/TB	47.5%	68.0%	54.4%
GLO	52.2%	64.8%	64.0%
PA	54.2%	41.6%	82.4%
ApoA-I	50.5%	63.2%	60.0%
ApoB	52.9%	36.8%	78.4%

### 3.4 Multivariable model construction and validation

**Figure 6:**
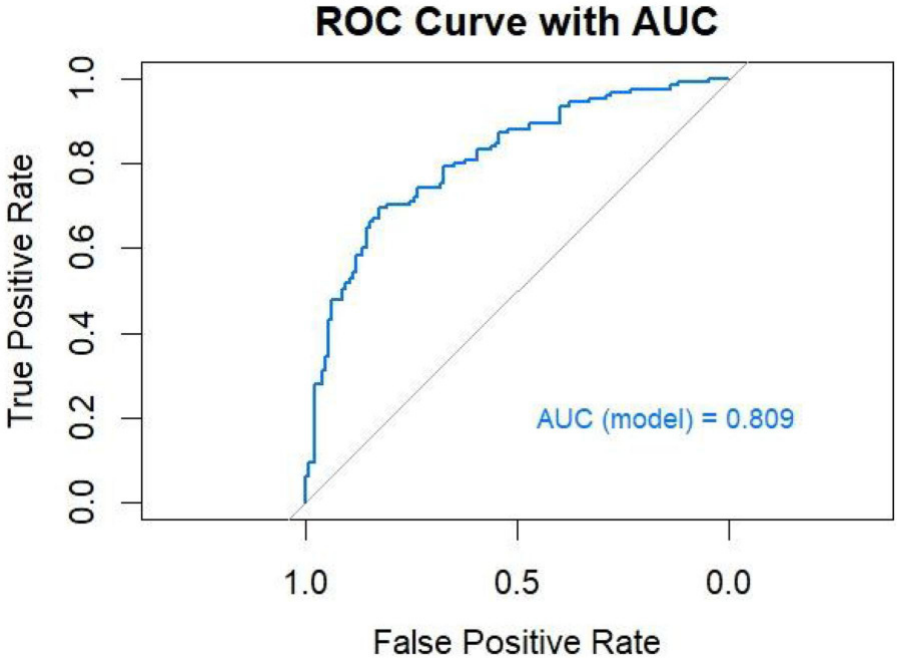
ROC curve of the multivariable model.

The ROC curve of the model constructed using multivariable binary logistic regression demonstrated excellent discriminative ability, with the curve lying entirely above the reference diagonal, yielding an AUC of 0.809 (Figure 6). The optimal threshold was found to be 56.8%, with a sensitivity of 69.6% and specificity of 82.4%. DeLong pairwise comparison results are shown in Table 3. The comprehensive model significantly outperformed each individual factor (ABSI, EH, DB/TB, GLO, PA, ApoA-I, ApoB) in terms of AUC (all *P* < 0.001). Pairwise comparisons between single factors showed no significant differences in most cases: for example, ABSI vs. GLO: *P* = 0.958, ABSI vs. DB/TB: *P* = 0.801, ABSI vs. EH: *P* = 0.102; EH vs. ApoB: *P* = 0.973; DB/TB vs. GLO: *P* = 0.764; PA vs. ApoA-I: *P* = 0.751; ApoA-I vs. ApoB: *P* = 0.290. The *P* values for the remaining comparisons are presented in the table.

**Table 3:** DeLong validation.

Variable	ABSI	EH	DB/TB	GLO	PA	ApoA-I	ApoB	Model
ABSI	1	0.102	0.801	0.958	0.424	0.649	0.128	<0.001***
EH	0.102	1	0.152	0.079	0.409	0.214	0.973	<0.001***
DB/TB	0.801	0.152	1	0.764	0.568	0.823	0.262	<0.001***
GLO	0.958	0.079	0.764	1	0.424	0.583	0.137	<0.001***
PA	0.424	0.409	0.568	0.424	1	0.751	0.458	<0.001***
ApoA-I	0.649	0.214	0.823	0.583	0.751	1	0.29	<0.001***
ApoB	0.128	0.973	0.262	0.137	0.458	0.29	1	<0.001***
Model	<0.001***	<0.001***	<0.001***	<0.001***	<0.001***	<0.001***	<0.001***	1

*** *P* < 0.001.

### 3.5 Nomogram

**Figure 7:**
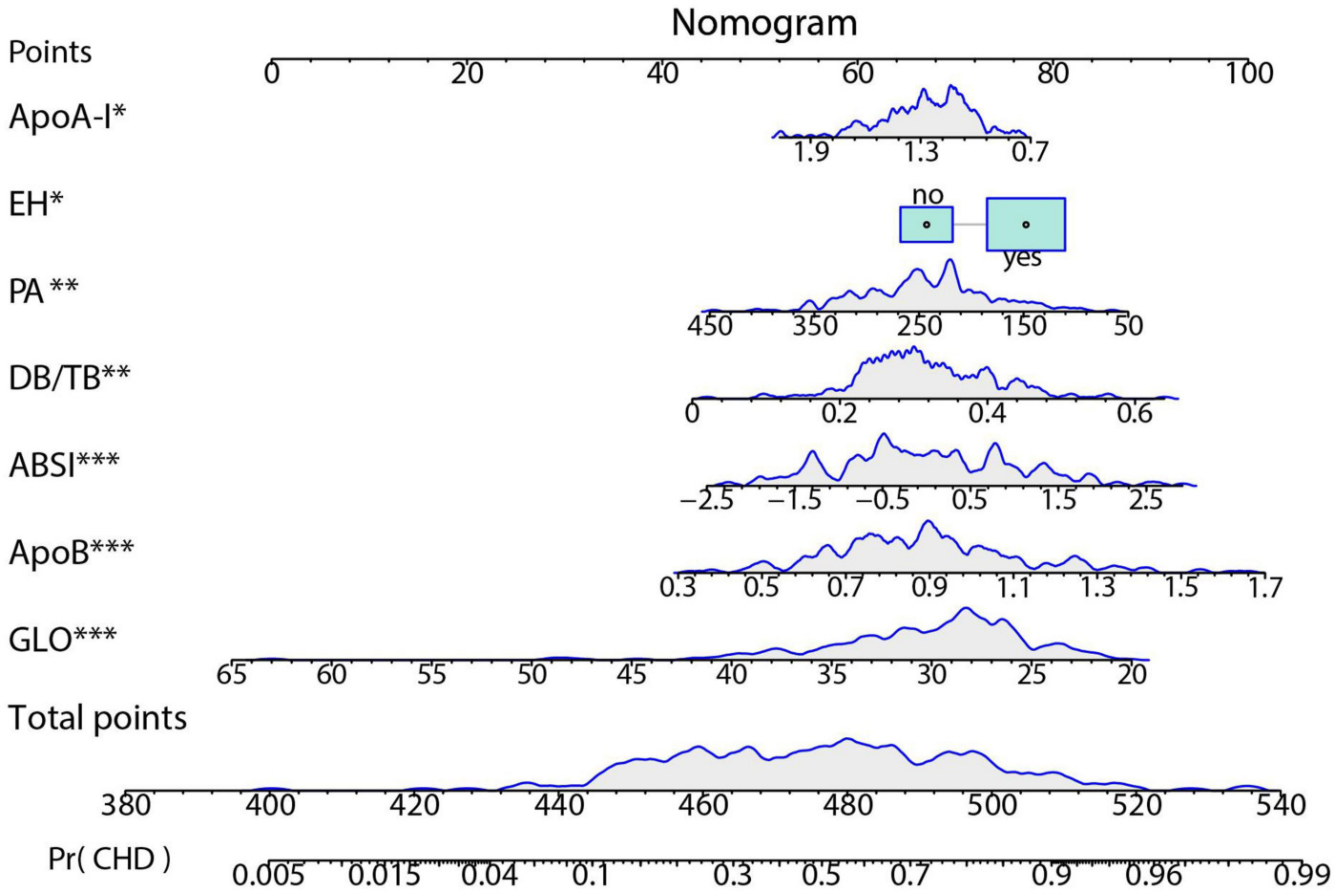
Nomogram for predicting CHD risk. The individualized risk should be read in the sequence of “variable value → Points → Total points → Pr(CHD)”. At the top is the Points scale (0–100). For each factor, locate its value on the corresponding axis and project upward to obtain the score; sum all scores to yield the Total points (approximately 380–540). Map the Total points downward to the bottom Pr(CHD) scale (0.005–0.99) to obtain the predicted probability. The blue density curves in the figure indicate the distribution ranges of each variable and the total score within the sample. ***P < 0.001; **P < 0.01; *P < 0.05.

A nomogram was constructed based on the final multivariable model to estimate the individual probability of CHD (Figure 7). The nomogram includes 7 predictor variables: ABSI, ApoA-I, EH, PA, DB/TB, ApoB, and GLO. The contribution of each variable to the total score was assessed by evaluating the scale range. ABSI, ApoB, and GLO exhibited the widest score ranges (*P* < 0.001) and contributed the most to the total score. PA and DB/TB were ranked next in terms of contribution (*P* < 0.01), while the contributions of ApoA-I and EH were relatively smaller (*P* < 0.05). EH is a binary variable (no/yes).

## 4 Discussion

In the PSM-matched case–control sample, LASSO regression, dynamic RFE, and random forest variable importance collectively identified a set of core predictors: ABSI, PA, DB/TB, ApoB, GLO, ApoA-I, and EH. Compared to a single predictor, a multivariable logistic regression model including these variables significantly improved CHD discrimination, suggesting that combined multifactorial evaluation is more conducive to risk identification. The nomogram derived from this model enables individualized risk estimation, with ABSI, ApoB, and GLO standing out as top contributors to the overall prediction.

### 4.1 Association of ABSI with CHD and underlying mechanisms

ABSI is a body shape index integrating waist circumference with height and BMI, aiming to capture abdominal (especially visceral) fat load more sensitively, and it provides a good representation of central obesity (8). Central obesity promotes atherosclerosis development via pathways including insulin resistance, an atherogenic lipid profile (high triglycerides and small, dense LDL with low HDL), elevated blood pressure, and systemic inflammatory responses, thereby increasing CHD risk (20, 21); free fatty acids and inflammatory factors (such as C-reactive protein) released by visceral fat can also accelerate this process (22). Meanwhile, abdominal obesity is closely related to non-alcoholic fatty liver disease (NAFLD), which further elevates CHD risk by exacerbating systemic inflammation and disturbances in lipid metabolism (23, 24). Previous studies are consistent with this: in Chinese normal-weight populations, ABSI is significantly associated with all-cause and cardiovascular mortality risk (16); different obesity indices (ABSI, waist-to-height ratio, body roundness index) show similar performance in predicting cardiovascular risk factors (17). In the present study, after PSM and multivariable adjustment, we found that ABSI was closely associated with CHD. Univariate analysis showed an OR of 1.757 (95% CI 1.345–2.332), *P* < 0.001. As a single index, its discriminative power was limited (AUC = 0.65), but sensitivity was high (86.4%) and specificity was low (40.0%), suggesting that ABSI is more suitable for screening and preliminary stratification. After integrating ABSI with ApoB, GLO, DB/TB, PA, ApoA-I, and EH, the model's discriminative ability improved significantly (AUC = 0.809; DeLong test *P* < 0.001 compared to any single index), and ABSI emerged as one of the top contributing predictors in the nomogram, underscoring its critical incremental value in a multi-indicator combined assessment.

### 4.2 Advantages of the multivariable model

Our multifactor model integrating ABSI, DB/TB, PA, GLO, ApoA-I, ApoB, and EH demonstrated significantly better discrimination than any single index (AUC = 0.809; DeLong tests vs. each single factor all *P* < 0.001), indicating that CHD risk assessment should involve a combined multi-factor evaluation. Each variable has a sound biological rationale. ApoB is the primary apolipoprotein of LDL, promoting atherogenesis; ApoA-I is the primary apolipoprotein of HDL and confers anti-atherosclerotic effects (25). The ratio of ApoB to ApoA-I is considered an even stronger risk marker (26). EH is a classic risk factor that can increase event risk through vascular wall stress and atherosclerotic pathways (27). Bilirubin has antioxidant properties, and an altered DB/TB ratio reflects changes in bilirubin metabolism or liver function status that are associated with increased risk (28). PA reflects inflammation and nutritional reserves; lower PA levels are independently associated with higher CHD risk and adverse outcomes (29–32). A reduced GLO indicates an impaired immune or nutritional status and is also linked to increased risk (33). By integrating ABSI, lipid profiles (ApoA-I/ApoB), bilirubin metabolism (DB/TB), inflammation/nutrition (PA, GLO), and blood pressure status (EH), our multi-dimensional model better captures the complex etiology of CHD and significantly improves the model's discriminative power and robustness.

### 4.3 Comparison with previous studies

Research has shown that in Chinese populations of normal weight, ABSI is significantly associated with cardiovascular mortality risk (16), a finding consistent with ours. Several Chinese studies suggest that socio-economic and lifestyle changes are driving the rising burden of ischemic heart disease (34), and that cardiovascular risk factors vary markedly by region (35). Comparative analyses of obesity-related indices indicate that ABSI, waist-to-height ratio and BRI have similar predictive performance (17). Modelling studies further underscore the contribution of long-term trends in blood pressure and lipid levels to CHD events (36); recent epidemiological data in China show continued increases in hypertension, dyslipidemia and diabetes (37). Compared with these studies, our work contributes by building a multivariable model that integrates ABSI, ApoB, GLO, DB/TB, PA, ApoA-I and EH, using a case-control design with PSM to ensure comparability. We visualized the model as a nomogram for individualized risk quantification. Additionally, BMI mainly reflects overall obesity and cannot differentiate fat distribution; therefore, its efficacy in predicting cardiovascular risk is weaker than that of indices reflecting central obesity (38). One large-sample analysis comparing various obesity metrics for CHD prediction showed that BMI's AUC was only about 0.56, whereas an optimized ABSI index reached approximately 0.73, markedly outperforming BMI (39). Another study noted that among individuals with normal BMI but excessive waist circumference (indicative of central obesity), the risk of cardiovascular death was even higher than among those classified as obese by BMI alone (40). This means many individuals with normal BMI yet excessive abdominal fat could be overlooked by traditional BMI assessments. Compared with the above studies, the incremental contribution of our study lies in the use of a PSM-matched case–control design to integrate seven factors (including ABSI) into a multivariable model and to develop a nomogram, achieving individualized risk quantification. The results demonstrate that ABSI plays a significant role in the combined model, providing a practical tool for CHD risk assessment in the Chinese context.

### 4.4 Limitations and future directions

This study has several limitations. First, as a single-center retrospective case–control study, it unavoidably carries selection bias, and the single-source sample limits the external generalizability of the findings. Second, even after PSM, the sample size remained relatively small (*n* = 250), raising the risk of model overfitting. The model's performance (e.g., AUC = 0.809) was evaluated using only internal validation and may therefore be overestimated. Furthermore, given the case–control design of this study, the observed associations should be interpreted with caution. Additionally, because certain potential confounding factors (such as medication use or lifestyle details) were not fully collected, residual confounding may have influenced the results. At the same time, each variable was measured only at a single time point, making it difficult to avoid the impact of measurement error and within-individual variability. Finally, the predictive model developed in this study has yet to undergo external validation in independent populations; its clinical applicability and external generalisability will remain uncertain until confirmed by multi-centre prospective studies. To address these limitations, future work should aim to validate the findings in larger and more diverse populations. Firstly, external validation can be conducted through prospective multi-centre cohort studies to evaluate the model's stability and applicability across different groups, thereby laying a reliable foundation for clinical practice. Importantly, before the model is used for clinical decision-making, its predictive performance and clinical value must be confirmed in independent cohorts. Secondly, future research can employ methods such as decision curve analysis (DCA) to assess the model's net benefit at different probability thresholds, thereby determining its clinical utility. In addition, further studies should explore the potential mechanisms linking indices like ABSI, DB/TB, and GLO to the development of CHD, evaluate the effectiveness of interventions targeting key risk factors, and compare the differences and commonalities of CHD risk factors among populations in different regions.

## 5 Conclusion

The present study identified ABSI as a potential predictor of CHD risk in a southern Chinese population, although its discriminatory power was limited when used alone. Incorporating ABSI into a multivariable model including DB/TB, PA, GLO, ApoA-I, ApoB and EH significantly improved predictive performance, and the nomogram derived from this model provides a preliminary framework for individualised CHD risk assessment; however, its clinical value still needs to be validated in independent prospective cohorts. While this study was limited by a single-centre sample, it provides a reference for CHD risk screening and intervention strategies in this population; thus, multi-centre studies are needed to verify the objectivity and generalisability of these results.

## Data Availability

The original contributions presented in the study are included in the article/Supplementary Material, further inquiries can be directed to the corresponding authors.
